# 1424. Modeling the Impact of Multi-Drug Resistant Organism (MDRO) Regional Containment Strategies in a Tennessee Patient Transfer Network

**DOI:** 10.1093/ofid/ofad500.1261

**Published:** 2023-11-27

**Authors:** Rany Octaria, Prabasaj Paul, Pamela Talley, Stephen Deppen, Peter F Rebeiro, Rachel Slayton, Marion Kainer

**Affiliations:** Centers for Disease Control and Prevention, Atlanta, GA; CDC, Atlanta, Georgia; Tennessee Department of Health, Nashville, Tennessee; Vanderbilt University Medical Center, Nashville, Tennessee; Vanderbilt University Medical Center, Nashville, Tennessee; CDC, Atlanta, Georgia; Western Health, Melbourne, Victoria, Australia

## Abstract

**Background:**

Centers for Disease Control and Prevention (CDC) guidance recommends coordinated multifacility interventions to contain selected MDRO, such as carbapenem-resistant Enterobacterales (CRE). A mathematical model using data from a northeastern state estimated these interventions would lead to 76% relative reduction in CRE prevalence in a hospital network connected through patient transfers, but whether results would be similar using data from another state is unclear.

**Methods:**

We used Tennessee (TN) surveillance and hospital discharge data to estimate CRE transmissibility and patient transfers in a deterministic compartmental model to simulate regional spread of CRE. Simulations were initialized with the first clinical detection of CRE in the hospital with the most outgoing transfers. Interventions were initiated in facilities that detected CRE and those sending or receiving the largest numbers of patients to/from these facilities, including: 1) biweekly point prevalence surveys; and 2) enhanced infection control implemented immediately on CRE detection. We assumed interventions reduced intrafacility transmission by 20%. Surveys stopped after 2 consecutive negative rounds in a facility (Figure 1). We ran simulations using 2 patient transfer networks: 1) all hospitalized patients; and 2) CRE surrogates (patients clinically similar to CRE-infected patients) (Figure 2).

**Figure 1.**
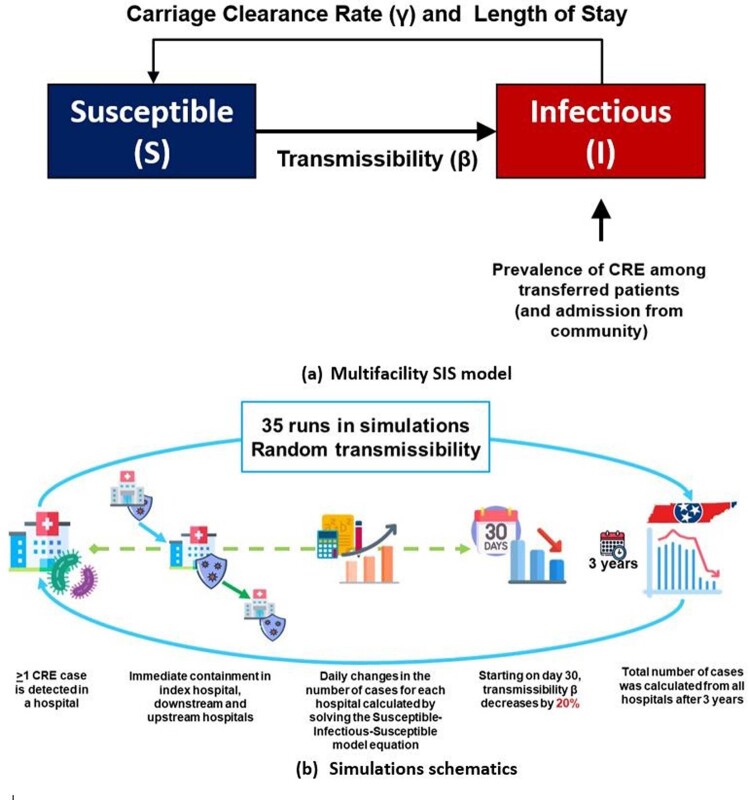
Methods: Multifacility Susceptible-Infectious-Susceptible model (a) and simulations schematics (b).

In the multi-facility SIS model, we assume hospitals are at constant occupancy. Each bed was occupied by either an infectious (I) or susceptible patient (S). (b) Interventions outlined in the simulations followed the recommendations of the Interim Guidance for a Public Health Response to Contain Novel or Targeted Multidrug-resistant Organisms (MDROs). Initial detection at the facility with the highest number of outgoing transfers followed by immediate containment efforts and reduced transmissibility in intervened hospitals by 20% by day 30. Simulations were run 35 times. Each run randomly selected a transmissibility value derived from the distribution in our estimates derived from CRE surveillance data in TN.

**Figure 2.**
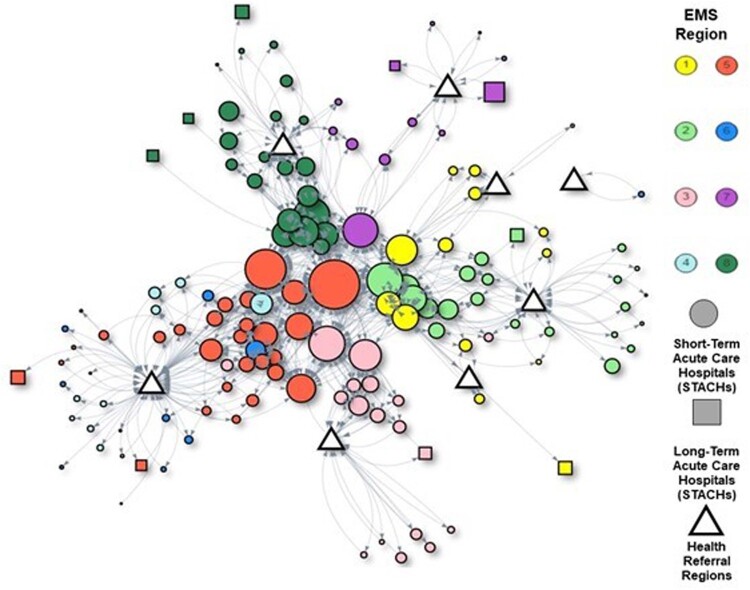
Methods: the patient transfer network of Tennessee (TN) hospitals and communities

The network of Tennessee (TN) hospitals and communities, represented by Health Referral Regions (HRRs) according to the Dartmouth Atlas of Healthcare (https://www.dartmouthatlas.org) connected by direct and indirect patient transfers with up to 365 days of intervening community stays. Patient transfer data were sourced from the 2018-2019 TN Hospital Discharge Database. Patients who were not admitted to another facility after being discharged in 2018 were attributed to transfers into the HRR corresponding to the hospital’s address. Each circle represents a short-term acute care hospital (STACH), each square represents a long-term acute care hospital (LTACH), and each triangle represents an HRR. The arrow represents the direction of transfers between hospitals. Node sizes correspond to the number of registered beds, and color represents the Emergency Medical Services (EMS) regions where the facility is located.

**Results:**

CRE case counts 3 years after importation and interventions were 21% lower (interquartile range [IQR]: 20–23%) than without interventions using the transfer network of all hospitalized patients; interventions were required in 52 (36%) of 144 hospitals. Simulations in the CRE surrogate network resulted in a 26% (IQR: 20–38%) CRE case reduction and interventions in 32 (24%) hospitals.

**Conclusion:**

The estimated CRE prevalence reduction achieved through simulating CDC guidance-recommended interventions in TN was lower than estimates using data from a different state, likely due to state-specific differences in patient transfer network structure and transmission parameters. Containment guided by transfer patterns of patients clinically similar to CRE-infected patients rather than all hospitalized patients requires interventions in fewer hospitals to achieve a comparable impact.

**Disclosures:**

**Peter F. Rebeiro, PhD, MS**, Gilead: Advisor/Consultant|Janssen Pharmaceuticals: Advisor/Consultant|National Institutes of Health: Grant/Research Support

